# Endometrial carcinosarcoma presenting with a solitary breast metastasis − A rare case report

**DOI:** 10.1016/j.gore.2025.101780

**Published:** 2025-06-10

**Authors:** Inês Taborda Leal, Maria Inês Barradas, Gonçalo Freitas, Filipa Castro Coelho, Filipa Santos, Mónica Cardoso, Maria Ana Serrado, Hugo Gaspar

**Affiliations:** a Department of Obstetrics and Gynecology, Hospital Doutor Nélio Mendonça, Av. Luís de Camões 6180, 9000-177 Funchal, Portugal; b Division of Gynecology Oncology Department of Obstetrics and Gynecology, Hospital Doutor Nélio Mendonça, Av. Luís de Camões 6180, 9000-177 Funchal, Portugal; c Department of Pathology, Hospital Doutor Nélio Mendonça, Av. Luís de Camões 6180, 9000-177 Funchal, Portugal; d Department of Radiology, Hospital Doutor Nélio Mendonça, Av. Luís de Camões 6180, 9000-177 Funchal, Portugal; e Department of Gynecology, Centro Hospitalar Universitário São João, Alameda Prof. Hernâni Monteiro, 4200-319 Porto, Portugal

**Keywords:** Endometrial carcinosarcoma, High-grade endometrial carcinoma, Breast metastasis, Atypical presentation

## Abstract

**Background:**

Endometrial carcinosarcoma (ECS) is a rare and highly aggressive malignancy, accounting for approximately 5% of all uterine cancers. While peritoneal seeding and pulmonary metastases are relatively common, breast involvement is exceedingly rare, with only a few cases reported in the literature, primarily involving the serous or clear cell subtypes and typically associated with widespread metastatic disease. To the best of our knowledge, this is the second documented case of a primary ECS metastasizing to the breast, and the first presenting as a solitary breast mass.

**Case Report:**

A 68-year-old woman presented to the emergency department with one week history of postmenopausal abnormal uterine bleeding. Endometrial biopsy confirmed the diagnosis of ECS. Staging thoracoabdominal computed tomography (CT) revealed a solitary 11 mm mass in the lower outer quadrant of the left breast, accompanied by two ipsilateral axillary lymph nodes, but otherwise no signs of distant metastases, additional lymphadenopathies, or peritoneal implants were described. Findings on abdominal and pelvic magnetic resonance imaging (MRI) were concordant with CT, demonstrating no suspicious lymphadenopathies or signs of distant metastatic disease. Breast biopsy revealed a papillary carcinoma with *CERB2* positivity. The patient underwent total hysterectomy, bilateral salpingo-oophorectomy, omentectomy, pelvic and lombo-aortic lymphadenectomy, as well as left breast lumpectomy and axillary lymphadenectomy. Complete cytoreductive surgery was performed with no macroscopic residual disease. Final pathology confirmed the breast lesioń metastatic origin.

**Conclusion:**

This case holds clinical significance as it underscores the necessity of considering a broad differential diagnosis when encountering uncommon or atypical presentations, particularly in high-grade malignancies such as ECS.

## Highlights

• Endometrial carcinosarcoma case presenting with an isolated breast lesion, without radiological evidence of metastasis.
• Awareness of atypical presentations of high–grade endometrial tumors.
• Critical role of histopathological evaluation in distinguishing metastasis from synchronous primary tumors.
• Multidisciplinary evaluation enabled accurate diagnosis and optimal treatment planning.

## 1 Introduction

Endometrial carcinosarcoma (ECS) is a rare biphasic malignancy composed of high-grade epithelial and sarcomatous components, accounting for approximately 5 % of all uterine cancers (Lopez-Garcia and Palacios, 2010, Matsuzaki, et al., 2021, Bogani, et al., 2023).

Risk factors for ECS include advanced age, prior pelvic radiotherapy, Black race, and conditions associated with hyperestrogenism, such as obesity, nulliparity, exogenous estrogen exposure, and tamoxifen use (Lopez-Garcia and Palacios, 2010, Singh, 2014). ECS predominantly affects postmenopausal women, during the sixth decade of life (Singh, 2014, Bogani, et al., 2023).

Endometrial cancer primarily spreads through direct extension and lymphatic pathways. Hematogenous dissemination, although less common, occurs more frequently in high-risk subtypes such as carcinosarcoma, resulting in distant metastases most commonly found in the lungs, liver, spleen, bone, and, rarely, the brain (AlHamaqi et al., 2022, Jaafar, et al., 2025). Metastasis to the breast remains exceptionally rare, representing less than 1 % of all breast tumors and can, therefore, be easily mistaken for primary breast cancer (Akçay, 2002, McEachron, et al., 2019). The most common malignancies that metastasize to the breast include melanoma, lung, gastrointestinal tract, and ovarian cancer (Akçay, 2002, Delair, et al., 2013).

Metastasis from uterine primaries to the breast has been reported in literature but are extremely rare (Farghaly, 2013, Tayson Lin, 2017, McEachron, et al., 2019, Choo et al., 2023). To date, this is only the second documented case of a primary ECS metastasis to the breast and the first presenting as a solitary breast lesion. AlHamaqi et al. described one case of a metastatic uterine carcinosarcoma presenting as an isolated breast mass, without concurrent intra-abdominal or other distant metastases; however, staging CT had previously revealed bilateral solid and cystic ovarian masses (AlHamaqi et al., 2022).

Occult metastases are frequently present at diagnosis, with up to 60 % of patients initially classified as having early-stage disease being upstaged after primary surgery, based on final pathological findings (Bogani, et al., 2023, Vintzileos et al., 2025).

ECS exhibits significantly more aggressive behavior than other high-grade endometrial carcinomas (HGEC), with a 5-year survival rate of approximately 30 %, compared to over 80 % in stage I HGEC (Bogani et al., 2023). Given its rarity, there is no universally accepted treatment consensus for ECS. In advanced stages, complete cytoreductive surgery is preferred whenever macroscopic disease can be fully resected, as this approach has been shown to improve survival outcomes (Toboni et al., 2021, Bogani, et al., 2023).

## 2 Case presentation

### 2.1 Initial presentation and management

A 68-year-old woman with good performance status presented to the emergency department with one week history of postmenopausal uterine bleeding (AUB). Her medical history included rectal adenocarcinoma, treated with chemoradiotherapy 8 years prior to presentation (complete response), along with hypertension and obesity. She had no family history of malignancy, had been menopausal for 17 years without prior bleeding, denied weight loss or breast symptoms, including previous history of breast lumps, skin discoloration or nipple discharge. Her screening mammogram 1 year prior to presentation was BIRADS 2.

**Fig. 1:**
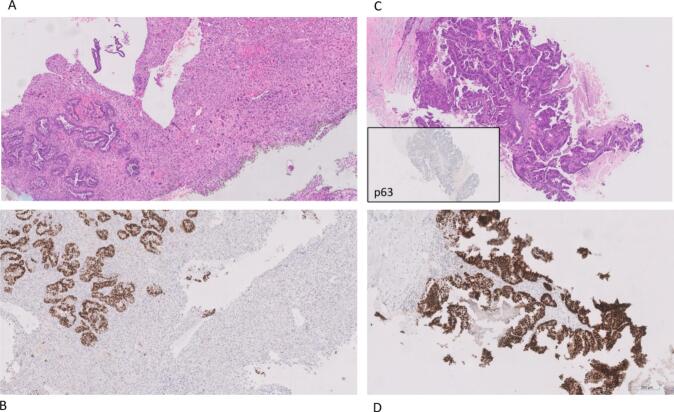
A. Hematoxylin and eosin (H&E) staining of the endometrial biopsy shows a biphasic tumor with a glandular component (left) and a mesenchymal component (right). **1B** – PAX8 immunohistochemistry highlights strong nuclear positivity in the glandular component (PAX8-positive) and negativity in the mesenchymal component (PAX8-negative). **1C** – H&E staining of the breast biopsy reveals a tumor with villoglandular/papillary architecture and absence of myoepithelial cells (p63-negative). **1D** – Immunohistochemical staining of the breast lesion shows PAX-8 positivity, supporting its uterine origin.

On gynecological examination, a polypoid mass protruding through the cervix was identified and biopsied. Transvaginal ultrasound demonstrated a 6 mm, richly vascularized endometrial thickening. The endometrial biopsy revealed a biphasic neoplasm with both, an epithelial adenocarcinoma and a solid mesenchymal (sarcomatous) component, the latter displaying pleomorphic neoplastic cells and necrosis. Immunohistochemistry showed a glandular component *PAX8*-positive, vimentin-negative, p16-positive, ER-positive (40 %) and a mesenchymal component *PAX8*-negative, vimentin-positive and ER-negative (Fig. 1A and B).

Evaluation of mismatch repair proteins showed intact expression of *MLH1, MSH2, MSH6*, and *PMS2*, indicating microsatellite stability. Aberrant *p53* expression was observed. These findings were consistent with a diagnosis of endometrial carcinosarcoma.

**Fig. 2:**
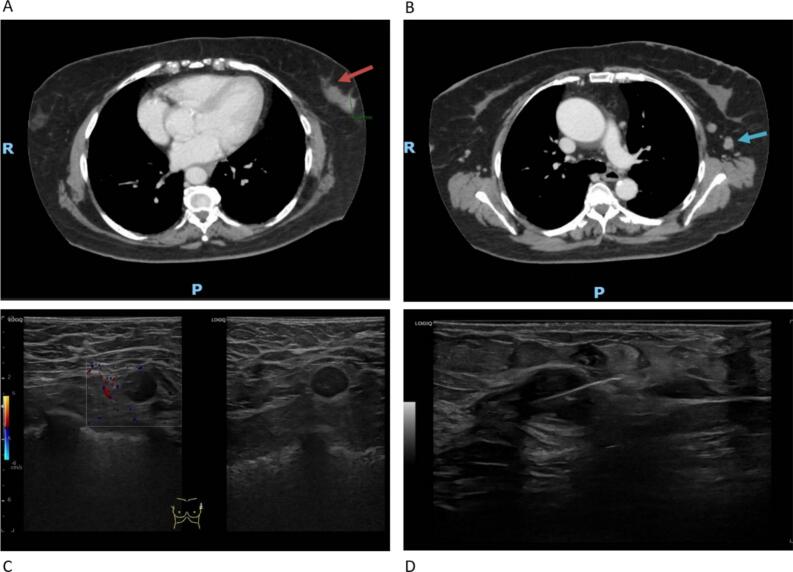
A. Axial contrast-enhanced CT (portal venous phase) shows an enhancing, slightly spiculated mass in the left breast (red arrow). **2B** – At a higher axial level, two round left axillary lymphadenopathies with cortical thickening are identified (blue arrow). **2C** – Ultrasound image shows the left axillary lymphadenopathies. **2D** – Ultrasound-guided needle biopsy of the left breast lesion. (For interpretation of the references to colour in this figure legend, the reader is referred to the web version of this article.)

The thoracoabdominal computed tomography (CT) performed for staging purposes identified a solitary 11 mm lesion in the lower outer quadrant of the left breast and two ipsilateral axillary lymph nodes (Fig. 2A and B).

No radiologic evidence of distant metastases in the lungs, liver, pancreas, gastrointestinal tract or bones were described. There were no morphological signs of inguinal, iliac, or obturator adenopathies, nor any suspicious lymph nodes in the upper abdominal or lumbo-aortic chains and no peritoneal implants were described. Additional findings included a mild dilation of the bile ducts and a small filling defect at the level of the ampulla, prompting further evaluation with magnetic resonance cholangiopancreatography, abdominal and pelvic magnetic resonance imaging (MRI).

**Fig. 3:**
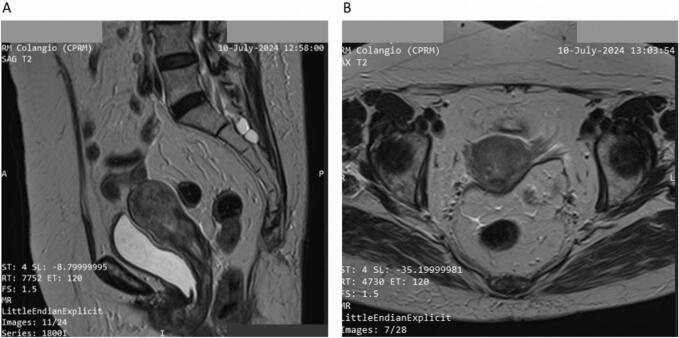
A. Sagittal T2-weighted MRI shows a tumor enlarging the endometrial cavity and protruding into the cervical canal. **3B** – Axial T2-weighted MRI demonstrates invasion of the tumor into the outer myometrial wall.

Pelvic MRI revealed a uterus measuring 7 x 5 x 4.5 cm, with loss of endometrial definition and diffuse invasion extending into the outer one-third of the myometrium and the cervix (Fig. 3A and B). No invasion of the vagina, bladder or adnexal regions was observed. Findings on abdominal and pelvic MRI were consistent with those of the CT, both demonstrating no evidence of suspicious lymphadenopathies or distant metastatic disease.

The case was reviewed by the senologic and the gynecologic oncology multidisciplinary tumor boards (MDTB) and a breast biopsy was ordered in order to clarify the origin of the breast tumor. The ultrasound-guided biopsy revealed a neoplasm with papillary architecture, lacking myoepithelial cells (p63-negative), ER-positive, PR-negative, *CK7-positive* and *HER2 (CERB2)* score 3+ (Fig. 1C). *PAX8* testing was unavailable at that time. Two possible diagnoses were considered: A) synchronous primary tumors (ECS and primary breast carcinoma); B) breast metastasis from ECS.

Given imaging findings suggestive of pelvis–confined disease and the patient’s good performance status, the MDTB decided for primary cytoreductive surgery, regardless of whether the lesions were synchronous tumors or breast metastasis with ECS. The patient underwent total abdominal hysterectomy, bilateral salpingo-opherectomy, infra-colic omentectomy, pelvic and *para*-aortic lymphadenectomy, as well as left breast lumpectomy and axillary lymphadenectomy. Intraoperatively, a bulky uterus, nodular omentum, and enlarged left *para*-aortic lymph node were observed, but no carcinomatosis or peritoneal surface involvement was noted. Complete debulking surgery with no macroscopic disease was achieved. Subsequent immunohistochemical analysis of the breast lesion showed *PAX8* positivity and *GATA3* negativity (Fig. 1D), which further supported the uterine origin.

### 2.2 Final pathology and disposition

Final pathologic examination of the postoperative specimen confirmed ECS, with a maximum tumor diameter of 6.5 cm and myometrial invasion exceeding 50 % of the uterine wall. The tumor involved the left fallopian tube, both ovaries, parametrium, omentum and *para*-aortic lymph nodes; peritoneal cytology was also positive. Multiple lymphovascular invasion foci were observed. The left breast lumpectomy specimen measured 2.1 × 1.2 cm and contained an invasive papillary neoplasm (p63-negative) with clear surgical margins; immunohistochemistry confirmed ECS origin. Of the 18 axillary lymph nodes retrieved, three were positive for metastatic disease.

The postoperative period was complicated by a surgical wound infection with multidrug-resistant organisms, which, in combination with the patientś obesity, delayed wound healing and prolonged her hospital stay. As a result, adjuvant chemotherapy could not be initiated within the recommended 12-week window. Subsequent management included hormonal therapy with a progestin. She remains under close oncological surveillance and is disease-free six months after surgery.

## 3 Discussion

Endometrial carcinosarcoma is a rare gynecologic malignancy that occurs almost exclusively in postmenopausal women, with a mean age at diagnosis of 60  years (Singh, 2014, Bogani, et al., 2023). Other important risk factors include prior pelvic radiotherapy, Black race, and hyperestrogenic states such as obesity, nulliparity, exogenous estrogen exposure and tamoxifen use (Lopez-Garcia and Palacios, 2010, Singh, 2014). In the presented case, the patient was a 68–year–old woman with a body mass index of 34 and a history of pelvic radiotherapy for rectal cancer, both significant risk factors for ECS.

The clinical presentation of ECS is non-specific, often resembling other endometrial carcinomas. Most frequently, symptoms include postmenopausal AUB, leukorrhea and/or abdominal pain, typically associated with a rapidly growing uterine mass (Singh, 2014, Bogani, et al., 2023). Endometrial biopsy is essential for prompt diagnosis, as demonstrated in this case.

Although rare, ECS accounts for a disproportionate high mortality due to its aggressive nature, high recurrence rate, frequent metastases at diagnosis and high potential for occult extra-uterine disease, which may not be detectable by pre-operative evaluation (Lopez-Garcia and Palacios, 2010, Bogani, et al., 2023, Vintzileos et al., 2025). In a large staging series, 56 % of patients presumed clinical stage I at diagnosis were upstaged following primary surgery, based on final pathology. Upstaging predominantly involved pelvic/para‐aortic nodes (Stage IIIC1/IIIC2) in 48,7% of cases and distant metastases (Stage IVB) in 25 % of cases (Vintzileos et al., 2025).

The metastatic behavior of endometrial carcinosarcoma is largely influenced by the predominant histological component of the tumor. Evidence suggests that the carcinomatous component typically determinates the clinical course of the disease. The epithelial (carcinomatous) component tends to metastasize through pelvic and para‐aortic lymphatic channels, whereas the mesenchymal (sarcomatous) component more often invades the peritoneal cavity or spreads hematogenously (Jaafar et al., 2025).

The pelvic/*para*-aortic lymph nodes, peritoneum and lungs, are common metastatic sites, whereas breast involvement is exceedingly rare (Akçay, 2002, AlHamaqi et al., 2022), accounting for < 1.0 % of all breast tumors (McEachron et al., 2019). In the uncommon event of metastasis to the breast, the upper-outer quadrant is most often affected due to its higher vascularity, suggesting hematogenous dissemination (AlHamaqi et al., 2022). Hematogenous metastases to the breast tend to be multiple and bilateral whereas lymphatic metastases usually present as solitary, well–circumscribed lesions with rapid growth in radiological images (Akçay, 2002, AlHamaqi et al., 2022). This patientś solitary left lower–outer–quadrant breast tumor and ipsilateral axillary lymphadenopathies—without other identifiable metastases in the most frequent anatomical places associated to ECS, led us to initially suspect of two synchronous neoplasia − ECS plus primary breast cancer (BC).

In fact, BC represents the most commonly diagnosed malignancy among women worldwide. In the context of this case, the initial suspicion of a primary BC would have been clinically plausible as initial breast biopsy findings (papillary architecture, ER–positive/PR–negative, *HER2* 3 + ) supported a breast primary. Lack of *PAX8* staining at that moment further complicated the differential diagnosis.

Histopathological and immunohistochemical analysis is critical to distinguish these entities (Lopez-Garcia and Palacios, 2010, McEachron, et al., 2019). In our case, subsequent staining revealed *PAX8* positivity and *GATA3* negativity in the breast lesion, strongly suggesting its uterine origin.

This case highlights the critical role of a multidisciplinary team—including gynecologic oncologists, senology team, oncologists, radiologists and pathologists— in the management of an endometrial carcinosarcoma with unusual presentation.

Due to its rarity and absence of large randomized trials, there is no established consensus on the optimal approach on ECS management (Bogani et al., 2023). However, ECS is now considered a primary endometrial carcinoma and so its treatment aligns with the guidelines for other non-endometrioid HGEC, which combines surgery, chemotherapy, and/or radiotherapy (Singh, 2014, Bogani, et al., 2023). Endocrine therapy might be considered in metastatic hormone-positive tumors, whenever chemotherapy is not feasible (Singh, 2014, Bogani, et al., 2023).

Nevertheless, complete cytoreductive surgery remains the cornerstone of treatment for non-metastatic or oligometastatic disease, whenever complete macroscopic resection is feasible with acceptable morbidity and quality of life outcomes, as it has been shown to significantly improve patient survival (Concin, et al., 2021, Matsuzaki, et al., 2021, Bogani, et al., 2023). In this case, surgery was chosen as the initial treatment, given that complete macroscopic resection was deemed achievable and best suited for this patient.

## 4 Conclusion

Endometrial carcinosarcoma is a highly aggressive malignancy with a marked propensity for early dissemination and high potential for occult extra-uterine disease. Although metastases to the breast from uterine primaries are exceptionally rare, it is essential to consider this possibility in the differential diagnosis when encountering atypical presentations.

Given the limited epidemiological data and the scarcity of high-quality evidence on ECS, further research is essential to better understand its behavior and guide evidence-based management strategies.

## 5 Patient consent

Written informed consent was obtained from the patient for the publication of this case report and accompanying images. A copy of the written consent is available for review by the Editor-in-Chief of this journal on request.

## Author contributions

ITL, FCC, MIB and GF collected data and contributed to the manuscript with, ITL being lead on the case report. FS, HG, MC and MAS supervised conceptualization and manuscript, production including and reviewing and editing the manuscript.

## CRediT authorship contribution statement

**Inês Taborda Leal:** Writing – original draft, Methodology, Investigation, Conceptualization. **Maria Inês Barradas:** Writing – review & editing. **Gonçalo Freitas:** Writing – review & editing, Conceptualization. **Filipa Castro Coelho:** Writing – review & editing, Investigation. **Filipa Santos:** Writing – review & editing, Supervision, Conceptualization. **Mónica Cardoso:** Writing – review & editing, Validation. **Maria Ana Serrado:** Writing – review & editing. **Hugo Gaspar:** Writing – review & editing, Validation, Supervision.

## Declaration of Competing Interest

The authors declare that they have no known competing financial interests or personal relationships that could have appeared to influence the work reported in this paper.
